# *PRECISION.array*: An R Package for Benchmarking microRNA Array Data Normalization in the Context of Sample Classification

**DOI:** 10.3389/fgene.2022.838679

**Published:** 2022-07-22

**Authors:** Huei-Chung Huang, Yilin Wu, Qihang Yang, Li-Xuan Qin

**Affiliations:** Department of Epidemiology and Biostatistics, Memorial Sloan Kettering Cancer Center, New York, NY, United States

**Keywords:** microRNA, microarray, normalization, classification, benchmarking

## Abstract

We present a new R package *PRECISION.array* for assessing the performance of data normalization methods in connection with methods for sample classification. It includes two microRNA microarray datasets for the same set of tumor samples: a re-sampling-based algorithm for simulating additional paired datasets under various designs of sample-to-array assignment and levels of signal-to-noise ratios and a collection of numerical and graphical tools for method performance assessment. The package allows users to specify their own methods for normalization and classification, in addition to implementing three methods for training data normalization, seven methods for test data normalization, seven methods for classifier training, and two methods for classifier validation. It enables an objective and systemic evaluation of the operating characteristics of normalization and classification methods in microRNA microarrays. To our knowledge, this is the first such tool available. The R package can be downloaded freely at https://github.com/LXQin/PRECISION.array.

## Introduction

Sample classification is an important goal in precision oncology for informing practitioners on treatment decisions and trialists on patient stratification (Pencina and Peterson, 2016; Pencina et al., 2020). Many classifiers that have been reported in the literature suffered irreproducibility partly due to data artifacts that result from disparate handling of tissue specimens (Simon et al., 2003; Ransohoff, 2005; Akey et al., 2007; Ioannidis et al., 2009; McShane et al., 2013). While data normalization is routinely used to circumvent the negative impacts of these artifacts, its performance has been evaluated primarily for differential expression analysis and is yet to be thoroughly assessed for the development of sample classifiers (Rahman et al., 2015; Qin et al., 2016).

To enable such an assessment, we utilized two datasets from the same set of tumor samples using Agilent microarrays for microRNAs (miRNAs), a class of small RNAs closely linked to carcinogenesis (Dillies et al., 2013; Maza et al., 2013). One dataset was collected with uniform handling and balanced array-to-sample assignment, and the other had the samples arrayed over time in the order of collection (Qin et al., 2014; Qin et al., 2018). To simulate addition paired datasets that mimic real-world data distribution, we used the first dataset to approximate the biological effects for each sample, serving as the “virtual samples”; we used the difference between the two arrays (one from each dataset) for the same sample to approximate the array effects for each array in the second dataset, serving as the “virtual arrays.” They can then be used for “virtual re-hybridization,” a re-sampling-based algorithm, to simulate data under various signal-to-noise ratios (Rahman et al., 2015; Qin et al., 2018). We have built an R package PRECISION.array, PaiREd miCrorna analysIs of molecular clasSificatION for microarrays (https://github.com/LXQin/PRECISION.array), for interested researchers to use for assessing their choice of normalization methods in combination with various methods for sample classifier training and validation under a chosen level of signal-to-noise ratio.

## Implementation

MiRNAs were profiled for 96 endometroid endometrial and 96 serous ovarian tumor samples twice. One dataset used uniform handling (by one technician in one batch) and balanced array-to-sample assignment (*via* blocking and randomization), and the other used neither (by two technicians in multiple batches with the arrays assigned in the order of tumor sample collection) (Qin et al., 2014; Qin et al., 2018). The data for a random subset of the miRNAs are included in the PRECISION.array package for demonstration purposes. The full datasets can be loaded from the PRECISION.array.DATA package (https://github.com/LXQin/PRECISION.array.DATA), where the first dataset can be called using the function *data.benchmark()* and the second using *data.test()*.

The uniformly handled dataset is used to approximate the biological effects for each sample by calling the function *estimate.biological.effect()*; the difference between the two arrays (one from each dataset) for a sample is used to estimate the array effects for each array in the non-uniformly handled dataset by calling the function *estimate.handling.effect()*. We will refer to the former as “virtual samples” and the latter as “virtual arrays.” For proof of principle, we use tumor type, endometrial *versus* ovarian, as the endpoint for classification. The level of biological signals can be adjusted by calling the function *reduce.signal()*; the extent of handling effects can be changed by calling the function *amplify.handling.effect()*.

The 192 virtual samples are split randomly (balanced by tumor type) in a 2:1 ratio to a training set and a test set; the 192 virtual arrays are split nonrandomly, with the first 64 and last 64 arrays in the order of array processing for the training set and the middle 64 arrays for the test set. Data are then simulated through “virtual re-hybridization” by reassigning arrays to samples and then summing the biological effects for a sample and the array effects for its assigned array by calling the function *rehybridize()*. The array-to-sample assignment can follow either a confounding or a balanced design (*via* blocking, randomization, and stratification), which can be the same or different for the training set and the test set. Data for the test set with sample effects only (i.e., without adding array effects) are used to assess the accuracy of a classifier and serve as the benchmark.

Data preprocessing consists of the following three steps: (1) log_2_ transformation; (2) normalization for training data and frozen normalization for test data (i.e., mapping the empirical distribution of each individual test set sample to the “frozen” empirical distribution of the normalized training data), with or without batch effect correction; and (3) probe-replicate summarization using the median.

Our package currently includes the functions for (1) three normalization methods for training data, namely, median normalization, quantile normalization, and variance stabilizing normalization, plus no normalization as a reference; (2) seven normalization methods for test data: the aforementioned three normalization, either for test data alone or frozen toward training data, and pooled quantile normalization (where the combination of training data and test data is quantile normalized), plus no normalization as a reference; (3) seven methods for classifier building: Prediction Analysis for Microarrays (PAM) (Tibshirani et al., 2002), logistic regression with the Least Absolute Shrinkage and Selection Operator (LASSO) penalty for variable selection (Tibshirani, 1996), Classification to Nearest Centroids (ClaNC) (Dabney, 2006), Diagonal Linear Discriminant (DLDA) (Dudoit et al., 2002), K-Nearest Neighbors (kNN) (Keller et al., 1985), Random Forest (Cutler and Stevens, 2006), and Support Vector Machine (SVM) (Noble, 2006); and (4) two methods for classifier validation, namely, cross-validation and external validation. The aforementioned methods for normalization and classification are chosen because of their popularity in the literature on transcriptomics data analysis. Our package can also accommodate additional methods chosen by the user *via* functions *uni.handled.simulate()* and *precision.simulate()*.

The overall goal is to assess the accuracy (measured as the proportion of misclassified samples) of a classifier across various normalization and classification methods and between the two validation methods, as well as the interactions among these three choices of methods. The full pipeline of the assessment is provided by the wrapper *precision.simulate.multiclass()*.

## Summary

In this study, we introduce an R package called PRECISION.array, which assesses the performance of data normalization methods in combination with various classification methods and and validation approaches under a number of sample-to-array assignment designs and a range of signal-to-noise ratios for miRNA arrays.

## Data Availability

Publicly available datasets were analyzed in this study. This data can be found here: https://github.com/LXQin/PRECISION.array.DATA.

## References

[B1] Akey J. M. Biswas S. Leek J. T. Storey J. D. (2007). On the Design and Analysis of Gene Expression Studies in Human Populations. Nat. Genet. 39, 807–809. 10.1038/ng0707-807 17597765

[B2] Cutler A. Stevens J. R. (2006). [23] Random Forests for Microarrays. Methods Enzymol. 411, 422–432. 10.1016/s0076-6879(06)11023-x 16939804

[B3] Dabney A. R. (2006). ClaNC: Point-And-Click Software for Classifying Microarrays to Nearest Centroids. Bioinformatics 22, 122–123. 10.1093/bioinformatics/bti756 16269418

[B4] Dillies M.-A. Rau A. Aubert J. Hennequet-Antier C. Jeanmougin M. Servant N. (2013). A Comprehensive Evaluation of Normalization Methods for Illumina High-Throughput RNA Sequencing Data Analysis. Briefings Bioinforma. 14, 671–683. 10.1093/bib/bbs046 22988256

[B5] Dudoit S. Fridlyand J. Speed T. P. (2002). Comparison of Discrimination Methods for the Classification of Tumors Using Gene Expression Data. J. Am. Stat. Assoc. 97, 77–87. 10.1198/016214502753479248

[B6] Ioannidis J. P. A. Allison D. B. Ball C. A. Coulibaly I. Cui X. Culhane A. C. (2009). Repeatability of Published Microarray Gene Expression Analyses. Nat. Genet. 41, 149–155. 10.1038/ng.295 19174838

[B7] Keller J. M. Gray M. R. Givens J. A. (1985). A Fuzzy K-Nearest Neighbor Algorithm. IEEE Trans. Syst. Man. Cybern. 15, 580–585. 10.1109/tsmc.1985.6313426

[B8] Maza E. Frasse P. Senin P. Bouzayen M. Zouine M. (2013). Comparison of Normalization Methods for Differential Gene Expression Analysis in RNA-Seq Experiments. Commun. Integr. Biol. 6, e25849. 10.4161/cib.25849 26442135 PMC3918003

[B9] McShane L. M. Cavenagh M. M. Lively T. G. Eberhard D. A. Bigbee W. L. Williams P. M. (2013). Criteria for the Use of Omics-Based Predictors in Clinical Trials. Nature 502, 317–320. 10.1038/nature12564 24132288 PMC4180668

[B10] Noble W. S. (2006). What Is a Support Vector Machine? Nat. Biotechnol. 24, 1565–1567. 10.1038/nbt1206-1565 17160063

[B11] Pencina M. J. Goldstein B. A. D’Agostino R. B. (2020). Prediction Models - Development, Evaluation, and Clinical Application. N. Engl. J. Med. 382, 1583–1586. 10.1056/nejmp2000589 32320568

[B12] Pencina M. J. Peterson E. D. (2016). Moving from Clinical Trials to Precision Medicine. JAMA 315, 1713–1714. 10.1001/jama.2016.4839 27115375

[B13] Qin L.-X. Huang H.-C. Begg C. B. (2016). Cautionary Note on Using Cross-Validation for Molecular Classification. Jco 34, 3931–3938. 10.1200/jco.2016.68.1031 PMC5477984 27601553

[B14] Qin L.-X. Zhou Q. Bogomolniy F. Villafania L. Olvera N. Cavatore M. (2014). Blocking and Randomization to Improve Molecular Biomarker Discovery. Clin. Cancer Res. 20, 3371–3378. 10.1158/1078-0432.ccr-13-3155 24788100 PMC4079727

[B15] Qin L. X. Huang H. C. Villafania L. Cavatore M. Olvera N. Levine D. A. (2018). A Pair of Datasets for microRNA Expression Profiling to Examine the Use of Careful Study Design for Assigning Arrays to Samples. Sci. Data 5, 180084. 10.1038/sdata.2018.84 29762551 PMC5952865

[B16] Rahman M. Jackson L. K. Johnson W. E. Li D. Y. Bild A. H. Piccolo S. R. (2015). Alternative Preprocessing of RNA-Sequencing Data in the Cancer Genome Atlas Leads to Improved Analysis Results. Bioinformatics 31, 3666–3672. 10.1093/bioinformatics/btv377 26209429 PMC4804769

[B17] Ransohoff D. F. (2005). Bias as a Threat to the Validity of Cancer Molecular-Marker Research. Nat. Rev. Cancer 5, 142–149. 10.1038/nrc1550 15685197

[B19] Simon R. Radmacher M. D. Dobbin K. McShane L. M. (2003). Pitfalls in the Use of DNA Microarray Data for Diagnostic and Prognostic Classification. JNCI J. Natl. Cancer Inst. 95, 14–18. 10.1093/jnci/95.1.14 12509396

[B20] Tibshirani R. Hastie T. Narasimhan B. Chu G. (2002). Diagnosis of Multiple Cancer Types by Shrunken Centroids of Gene Expression. Proc. Natl. Acad. Sci. U.S.A. 99, 6567–6572. 10.1073/pnas.082099299 12011421 PMC124443

[B21] Tibshirani R. (1996). Regression Shrinkage and Selection via the Lasso. J. R. Stat. Soc. Ser. B Methodol. 58, 267–288. 10.1111/j.2517-6161.1996.tb02080.x

